# Characterization of an Ellipsoidal Radiometer

**DOI:** 10.6028/jres.108.011

**Published:** 2003-04-01

**Authors:** Annageri V. Murthy, Ingrid Wetterlund, David P. DeWitt

**Affiliations:** Aero-Tech, Inc., Hampton, VA 23666; SP Swedish National Testing and Research Institute, Boras, Sweden; National Institute of Standards and Technology, Gaithersburg, MD 20899-8441

**Keywords:** heat flux, radiometer, sensors

## Abstract

An ellipsoidal radiometer has been characterized using a 25 mm variable-temperature blackbody as a radiant source. This radiometer is intended for separating radiation from convection effects in fire test methods. The characterization included angular response, responsivity, and purge-gas flow effect studies. The angular response measurements showed that the reflection from the radiometer cavity was higher on one of the cavity halves relative to the other half. Further development work may be necessary to improve the angular response. The responsivity measured with reference to a transfer-standard electrical-substitution radiometer showed dependence on the distance of the radiometer from the blackbody cavity. The purge-gas had the effect of reducing the signal output nearly linearly with flow rate.

## 1. Introduction

Nils-Erik Gunners first proposed the ellipsoidal radiometer^1^ concept in 1967 [1]. The radiometer consists of an ellipsoidal cavity with a highly reflective (gold plated) surface. The aperture opening is located at one focus and the sensing element, typically a thermopile sensor, is located at the other focus. The radiometer has applications in furnace and flame radiation measurements since the sensing element is not directly exposed to the hot gases. In contrast to total flux sensors, like Gardon and Schmidt-Boelter gages, the ellipsoidal radiometer output is not sensitive to convection heat-transfer effects.

The proposed International Standard for calibration of heat-flux sensors used in fire testing [2] by the International Organization for Standardization (ISO) recommends the use of an ellipsoidal radiometer in the calibration chain. An ellipsoidal radiometer and a total-flux sensor, both calibrated in a primary vacuum facility, serve as primary standards to calibrate secondary-level sensors for both radiation and convection for a specified fire-test configuration.

Unlike open-type sensors, which normally have a cosine angular response, the ellipsoidal radiometer angular response depends on the reflective property of the cavity surface. An exploratory study at the National Institute of Standards and Technology (NIST) with a commercial ellipsoidal radiometer showed that the responsivity could be a function of the view-angle when viewing finite aperture blackbodies [3]. Corrections to account for the view-angle effects have been suggested [4]. Efforts to develop radiometers with improved angular response are in progress at SP^2^, for use in the ISO heat flux standard.

Recently, NIST performed an evaluation of a new ellipsoidal radiometer, which is under development by Nils-Erik Gunners in cooperation with SP. This study was in support of the SP research on ellipsoidal radiometers for the proposed ISO standard, and also to understand issues related to heat-flux sensor measurements. The evaluation, performed in the 25 mm variable temperature blackbody facility (VTBB), consisted of angular response, transfer calibration and purge-gas effect studies. This report describes the details of the investigation and the measured characteristics of the radiometer.

## 2. Radiometer

**Fig. 1:**
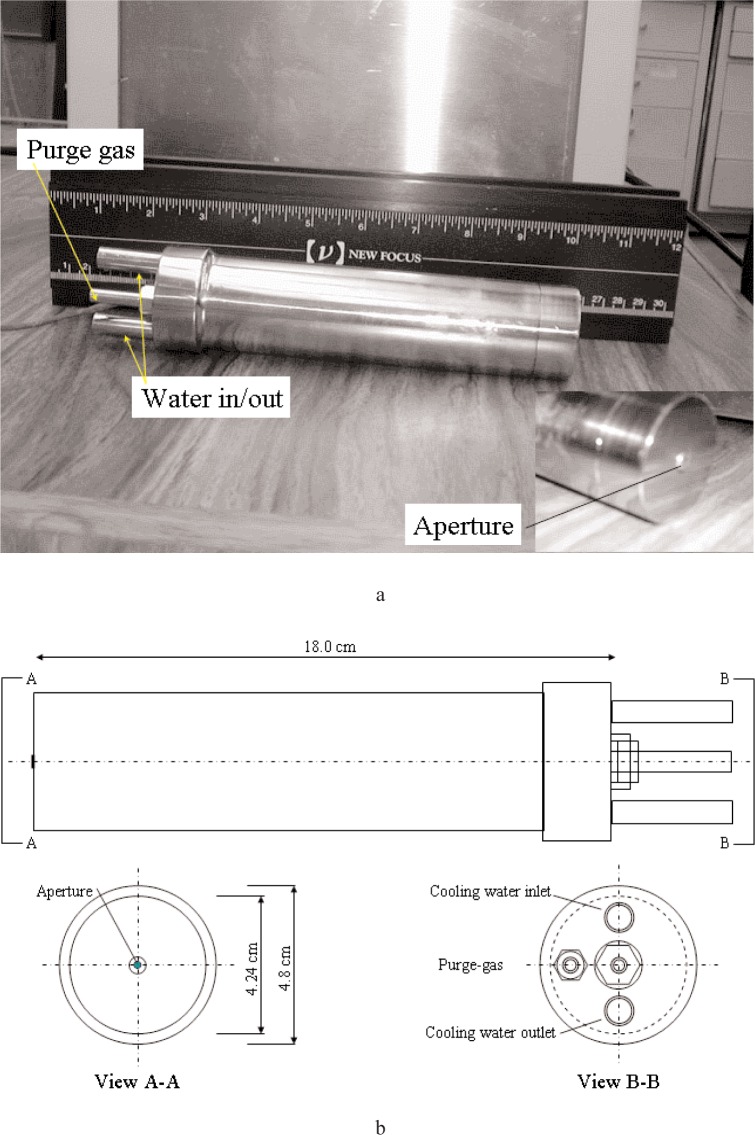
Ellipsoidal radiometer GR-2001-01 vacuum test version.

Figure 1 shows the test version of the radiometer [5]. The body diameter is 42 mm and length is 210 mm. The instrument is comprised of a receiving aperture, a gold-plated ellipsoidal cavity, and a thermopile sensor. The assembly is water-cooled and has provision for purging with an inert gas to prevent gases entering the cavity when the radiometer is used in a hostile environment. The manufacturer recommended a rather high cooling water flow rate of up to 0.4 L/s, particularly when operating in environments with high heat flux to the radiometer body. However, in the present tests a flow rate of 0.05 L/s was used. It was observed in the SP tests that the cooling water flow rate did not have significant effect on the radiometer output. The aperture receives radiation over a hemispherical field of view, and does not have any protective window. The gold-plated aperture projects about 0.7 mm through the hole in the front cap to ensure that the incident radiation is fully captured. The ellipsoidal shaped cavity surface thus ends in a sharp edge, ensuring maximum reflection of the radiation.

## 3. Test Facility

**Fig. 2:**
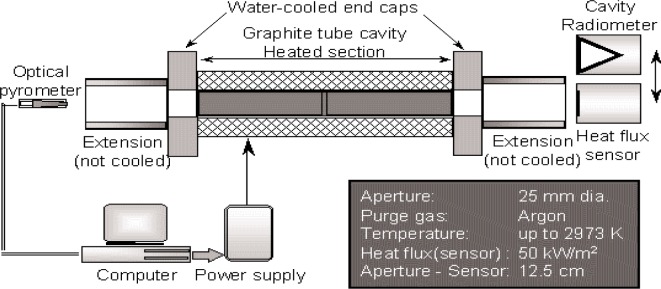
Schematic layout of the NIST variable temperature blackbody (VTBB).

The radiometer was characterized using the 25 mm VTBB as the radiant source. The VTBB is a primary facility used in radiance temperature calibrations. It has a large aperture and is particularly suitable for calibrating heat-flux sensors. The facility, shown schematically in Fig. 2, has been extensively used to calibrate heat-flux sensors and to study problems related to heat-flux sensor calibration using blackbody radiation. It is a thermally insulated and electrically heated graphite tube cavity. The heated tube cavity diameter is 25 mm and the heated section is 28.2 cm long with a central 3 mm thick partition. The tube end caps are water-cooled and are directly connected to the heating electrodes. The design provides a sharp temperature gradient between the end cap and the graphite heater element, and helps in minimizing the temperature gradients along the cavity length of the graphite tube. The end of the cavity-heating region is considered as the blackbody effective aperture for referencing distances. Two different lengths of graphite extension tubes can be attached to the end caps. The shorter extension is mounted when calibrating at higher heat flux levels, up to 50 kW·m^–2^. The extension tubes are not water-cooled.

An optical pyrometer measures the blackbody temperature by sensing radiation from one end of the furnace. A proportional-integral-differential (PID) controller regulates the power supply to maintain the furnace temperature to within ± 0.1 K of the set value. The maximum recommended operating temperature for the furnace is 2973 K.

For transfer calibration [6], the reference electrical substitution radiometer (ESR) and the sensor are located at a fixed distance away from the exit of the blackbody. At a distance of 12.7 mm from the exit, the maximum heat-flux is approximately 50 kW⋅m^–2^ to 60 kW⋅m^–2^. When calibrating at lower heat-flux levels of up to about 10 kW⋅m^–2^, the sensor and the radiometer are located at a distance of about 60 mm from the exit.

## 4. Test Description

**Fig. 3:**
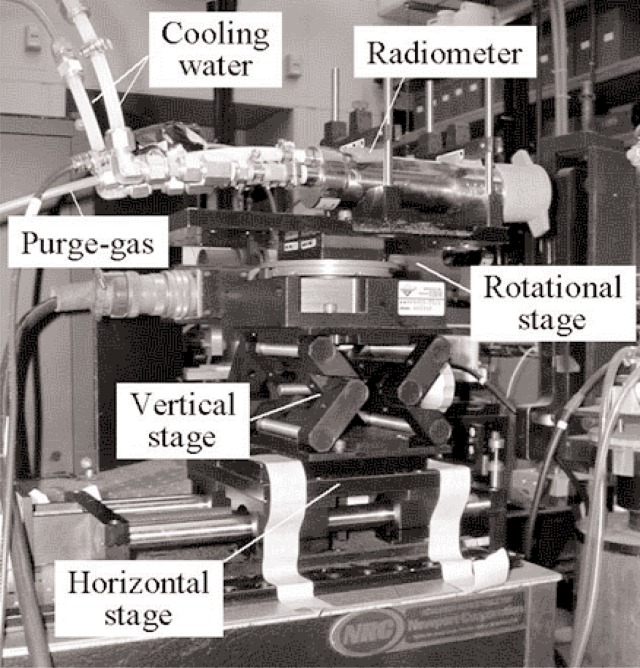
Radiometer setup arrangement in the 25 mm VTBB facility.

**Fig. 4:**
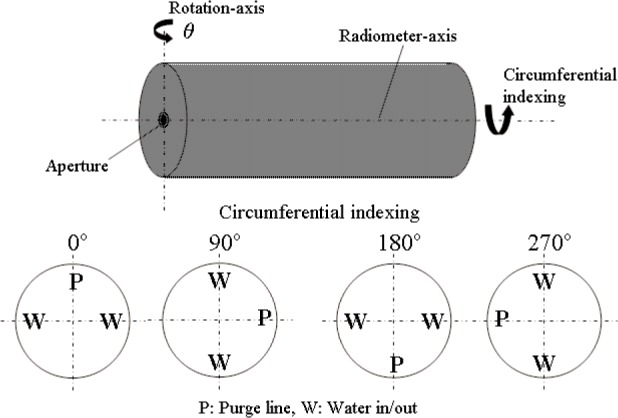
Rotational and circumferential indexing for angular response.

The primary objective of the tests in the VTBB was to measure the angular response of the radiometer. For this purpose, the radiometer was positioned in front of the blackbody with the radiometer axis normal to the radiating aperture of the blackbody. The radiometer axis was aligned with the blackbody axis using an alignment laser. A computer controlled rotational stage was used for angular indexing of the radiometer axis with reference to the blackbody aperture. The relative angular positioning uncertainty was better than 0.01°. The axis of rotation was aligned mechanically to be in the vertical plane and passing through the center of the radiometer aperture. The radiometer/rotational stage was mounted on a vertical stage for alignment with the blackbody axis. The complete assembly was mounted on a horizontal translation stage to perform measurements with the radiometer aperture in different vertical planes away from the blackbody. Figure 3 shows the experimental setup of the radiometer. Since it was speculated that the reflection from the radiometer cavity may not be uniform circumferentially, the angular response measurements were performed at four different angular positions by rotating the radiometer about its axis, as shown in Fig. 4. The blackbody was operated at a fixed temperature of 2773 K for all the angular response measurements.

The second objective of the test was to measure the responsivity of the radiometer with its axis aligned along the blackbody axis, and exposing the radiometer to varying levels of heat-flux by operating the blackbody at different temperatures. This measurement is a standard transfer calibration performed regularly in the VTBB facility. A cavity-type electrical substitution radiometer is used to determine the heat-flux level incident at the ellipsoidal radiometer aperture plane. The transfer calibration tests were performed with the radiometer located in three different planes away from the blackbody.

The VTBB tests in the open-air mode [6] make it ideal to evaluate the purge-gas effect on the radiometer response, which is otherwise difficult to perform in closed-mode or vacuum blackbody facilities. So, the last part of the test was to measure the effect of purge-gas flow rate on the radiometer output. The purge-gas outlet of the radiometer was connected to an argon compressed gas cylinder fitted with a pressure regulator and flow meter. The flow rate was varied in the range from 0 m^3^⋅s^–1^ to 14.2 × 10^–3^ m^3^⋅s^–1^ in 2.4 × 10^–3^ m^3^⋅s^–1^ steps, while operating the blackbody at a fixed temperature to maintain constant heat-flux level at the radiometer. The radiometer was at a fixed distance from the blackbody and normal to the radiating aperture. The blackbody was operated at two temperatures, 2273 K and 1873 K, corresponding to about 19 mV and 8 mV radiometer output at 0° incidence, with no purge-gas flow.

## 5. Results and Discussion

The following sections present the results for angular response, transfer calibration and purge gas effects along with a discussion of the implication of the results.

### 5.1 Angular Response

The angular response measurements were made at two locations of the radiometer, 0.268 m and 0.684 m, from the blackbody aperture. The signal levels at both positions were large enough compared to the background level. Initially, a measurement was performed with the radiometer and blackbody aligned collinear, corresponding to 0° angle-setting. Then, the radiometer was rotated through the range 0° to –90° in suitable steps taking a reading at each step. The radiometer was rotated back to 0° position and a check measurement was made. The measurement duration was about 10 s to 15 s at each angle setting. The same procedure was followed for measurements in the 0° to +90° angle range.

**Fig. 5:**
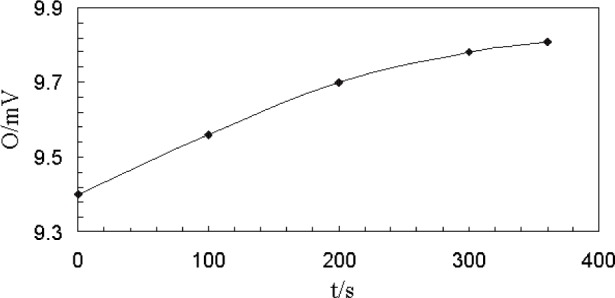
Radiometer output O in units of mV as a function of time *t* in seconds. (Position: 0.268 m from blackbody aperture, angle 0°).

Preliminary tests showed that the radiometer output, when rotated back to 0° position after rotating through 90°, differed from the initial reading. Also, the radiometer output when positioned at –90° or +90° angle was not zero. This behavior was speculated to be due to slow heating of the radiometer protruding-aperture while the measurements were being made over the angles from 0° to ±90°. This effect was confirmed by observing the radiometer output over a longer duration, shown in Fig. 5, when initially positioned at 0°. The gradual heating of the aperture increased the output level by about 4 % over a period of about 300 s. The increased output remained nearly constant when the radiometer was rotated over the range up to 90°. The output of the radiometer agreed with the initial reading when rotated back to 0° position. Therefore, to account for the aperture heating, the signal measured at 90° was used as a correction factor. The measured output at other angles was contracted by this factor.

**Fig. 6:**
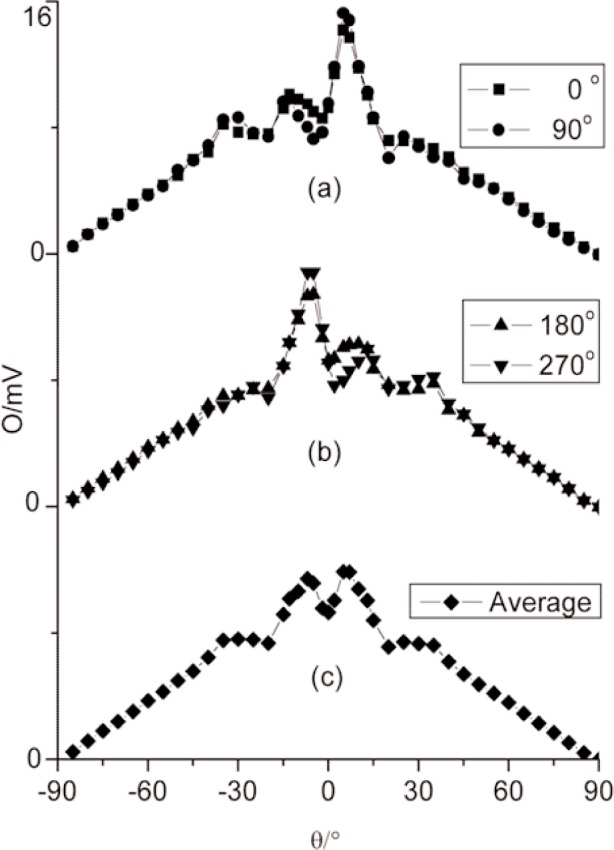
Radiometer output O in units of mV as a function of angle θ for different circumferential indexing, at location 0.268 m from the blackbody aperture.

Figures 6a–b show the corrected radiometer output for angle-settings in the range –90° to +90° when the radiometer positioned at a distance of 0.268 m from the blackbody aperture. The nominal heat-flux level at the location of the radiometer aperture was 7.2 kW⋅m^–2^. It may be observed from the distributions that the response is not symmetrical about 0° for all the radiometer orientations when rotated about its axis by 0°, 90°, 180°, and 270°. Sharp variations in signal level were observed for small departures from the 0° position. This suggests that the reflectance of the radiometer cavity surface is probably non-uniform leading to higher signal levels when the rays hit the sensor after reflection from one of the halves, and lower when turned by 180°. However, at angles beyond ±40°, the response is symmetrical about the 0° position. The non-uniformity and the anti-symmetrical nature of the distribution are limited to angles between ±40° with reference to radiometer axis. This behavior needs to be corrected in the next version to make the radiometer application suitable in situations when the view angle is within this range. It is likely that a local defective spot observed on the gold-plated cavity surface was the reason for the observed asymmetrical response. The effect of non-uniform reflectance of the radiometer cavity surface can somewhat be accounted by considering the average over different circumferential settings of the radiometer. The averaged output from measurements at 0°, 90°, 180°, and 270° positions is shown in Fig. 6c. This average of radiometric response is symmetric about the radiometer axis.

**Fig. 7:**
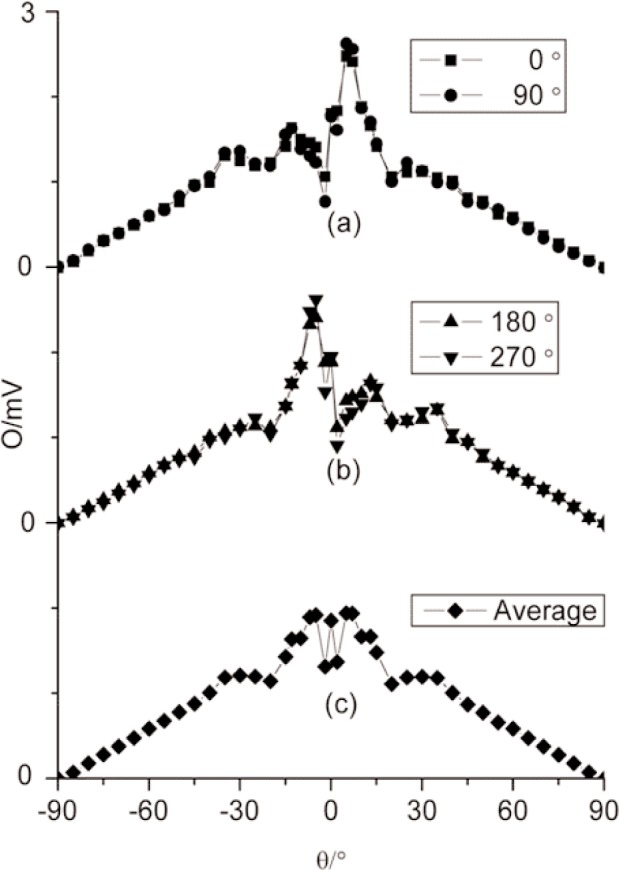
Radiometer output O in units of mV as a function of angle θ for different circumferential indexing, at location 0.684 m from the blackbody aperture.

A second set of measurements for the angular response was performed at a lower value of incident heat-flux level by locating the radiometer aperture-plane at a distance of 0.684 m from the blackbody aperture. The heat-flux level at this location was 1.2 kW⋅m^–2^. Figures 7a–c show the results of these measurements. The results are similar to the angular response observed when the radiometer was positioned at 0.268 m from the blackbody. The response average is again symmetrical about the radiometer axis.

**Fig. 8:**
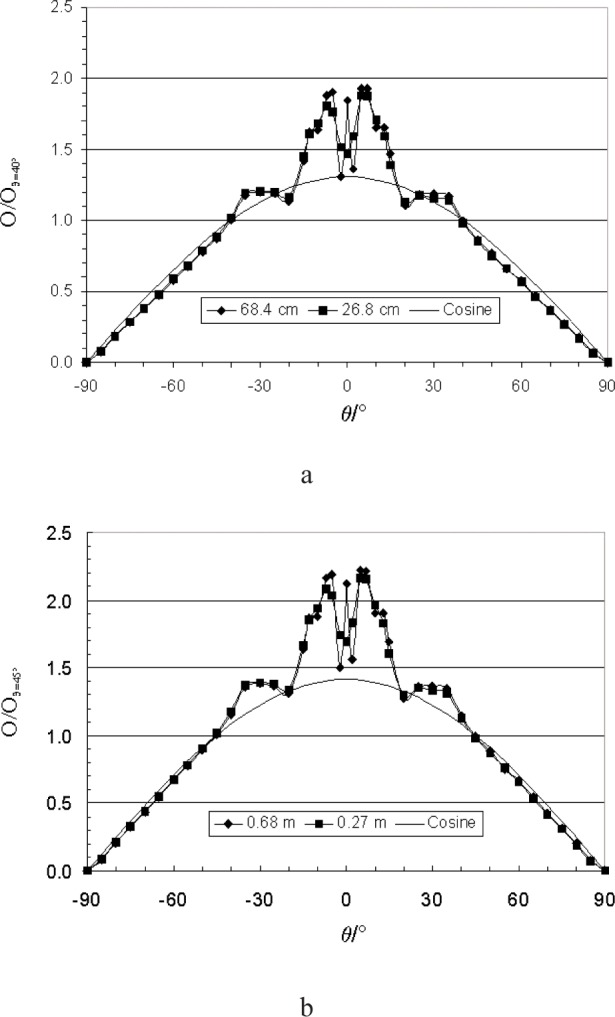
Mean radiometer output compared with cosine response. Output normalized with respect to average values at a) –40º and +40º, b) –45º and +45º.

The average response at the two positions, 0.268 m and 0.684 m, are shown normalized in Figs. 8a–b along with the cosine distribution. Figure 8a shows the normalized response distribution with reference to the average value at –40° and +40°. The distributions for both the radiometer positions overlap, and the agreement with the cosine distribution for angles beyond ±40° is good. The agreement is better when the average value of output at angles –45° and +45° positions is used as reference for normalizing (Fig. 8b). These comparisons represent qualitative angular response of the radiometer. The choice of using the values at ±40° or ±45° for normalizing is rather arbitrary. When the measured angular response is to correct for departure from the cosine behavior, the choice for a suitable reference needs to be examined further.

An ideal ellipsoidal radiometer absorbs the entire radiation incident at the aperture, which is located at one focus of the ellipsoid, and focuses it on the sensor located at the other focus. Thus the response of such an ideal radiometer should in principle be the same as that of the open sensor installed at the focus. An open sensor has nearly a cosine angular response. Differences from the ideal behavior happen due to non-focusing of the incident radiation by the cavity surface to the sensor location. Also, because of the imaging characteristics of the reflecting cavity surface, any radiation not focused at the sensor will escape, causing aperture loss. An appropriate choice for reference to correct for the non-cosine behavior appears to be the open sensor responsivity of the radiometer's thermopile sensor. Hence, it is desirable to have the characteristics of the open sensor determined before installation inside the cavity of the radiometer. Having as small an aperture as possible can reduce aperture loss.

It must be noted that in the present measurements the radiometer aperture is exposed to a finite source and the view-angle is rather narrow. Hence, the output of the sensor near 0° position is largely determined by radiation received directly from the source rather than that due to radiation reflected from the cavity surface. This situation is probably the cause for the observed large gradients in response for small changes in the angle about the radiometer axis. While this behavior is expected, the magnitude of the gradients appears rather large. The severity of such gradients will be of less significance when the incident radiation is hemispherical. Another possible approach to reduce the severity of the gradients in response is to increase the radiometer aperture size. Larger aperture diameter will promote in reflection from a larger portion of the cavity surface. However, this may result in a higher correction due to large aperture loss. But, it will likely result in a radiometer with a gradually varying angular response more amenable to empirical correction or calibration.

### 5.2 Transfer Calibration

**Fig. 9:**
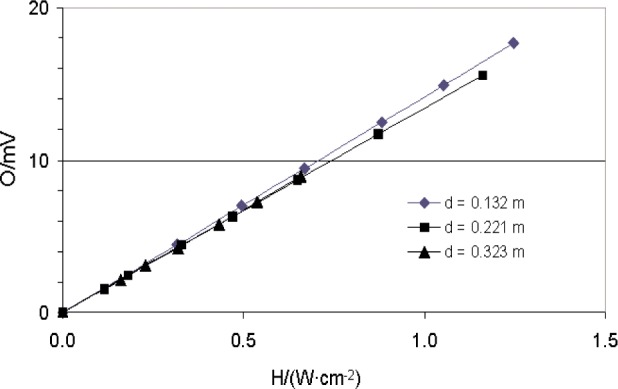
Radiometer output O in units of mV as a function of incident heat flux H in W⋅cm^–2^.

The radiometer responsivity was measured using the transfer calibration against a reference electrical substitution radiometer for three different positions of the radiometer: 0.132 m, 0.221 m and 0.323 m, respectively, from the blackbody aperture. For each position, the blackbody temperature was varied to obtain different heat-flux levels. Figure 9 shows the measured response of the radiometer at these locations. The response is linear over the range of calibration for all the three positions of the radiometer with respect to the blackbody.

**Fig. 10:**
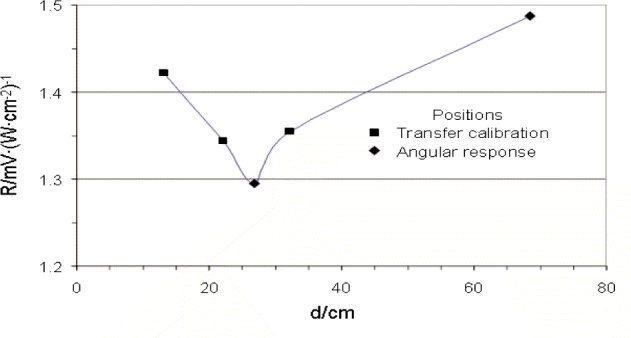
Radiometer responsivity *R* measured at different distances d from the blackbody aperture.

Table 1 gives the result of the linear regression analysis to the experimental data. The calculated regression factors were unity. The intercepts are also small suggesting negligible convection effects on the calibration. The responsivity at locations, 0.268 m and 0.684 m, obtained from a single data point at a blackbody temperature of 2773 K is also included in the Table. Figure 10 shows the variation of the responsivity with respect to the location from the blackbody. The measured responsivity decreases with distance initially by about 6 %. This trend is similar to that observed in reference [3]. However, when the radiometer is moved farther away from the blackbody, the responsivity showed a gradual increase.

While comparing the present transfer calibration results with other methods, it must be noted that large differences can exist due to non-cosine response of the ellipsoidal radiometer. These differences can be attributed to variations in the calibration methods rather than the radiometer itself. Responsivity measured under wide view-angle or hemispherical viewing conditions can be substantially lower than the present calibration performed under narrow view-angle conditions. These arguments reinforce suitability of the ellipsoidal radiometers under wide-view angle conditions for which they are primarily designed.

### 5.3 Purge Gas Effect

**Fig. 11:**
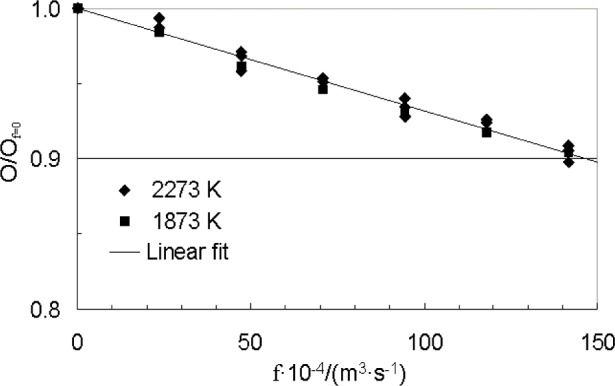
Radiometer output as a function of purge-gas flow rate *f*, normalized with respect to output at zero flow rate *f*_o_.

Figure 11 shows the influence of varying the purge-gas flow rate for two operating conditions of the blackbody temperature, 2273 K and 1873 K. The corresponding nominal output of the radiometer under these conditions was about 19 mV and 9 mV, respectively. The flow rate was varied up to about 0.015 m^3^/s for study study purposes. At the higher output level of 19 mV, measurements were made with both increasing and decreasing flow rates. Within the data scatter, the radiometer output decreases nearly linearly with flow rate. At the maximum flow rate, the decrease in signal output is about 10 % at both 19 mV and 9 mV output levels. The flow rates to be used in a practical application will be much less. The results of this experiment show that the flow rate for this radiometer should be kept less than 0.001 m^3^/s to keep the decrease in output to less 1 %.

## 6. Measurement Uncertainties

The measurement uncertainties associated with the transfer technique calibration in the 25 mm VTBB are discussed in references [6]. Table 2 lists the various factors affecting the uncertainty and the associated values. The uncertainty in the transfer standard (ESR) from the primary radiometric standard is based on previous measurements and is expected to be about 0.6 %. Items (b)–(i) show the uncertainties associated with transferring calibration from the ESR in the VTBB. The temperature of the VTBB during the test was steady to within about 0.1 K of the set value. The output of the ESR and the test radiometer were recorded for about 30 s to 60 s at all heat flux levels. The standard deviation of the mean of the readings was found to be within about 0.1 % of the mean value. The uncertainty due to alignment is less than 0.4 %, since a fixed length gage block was used to position the instruments in front of the blackbody. Item (j) represents the uncertainty to account for long-term repeatability. This value of the uncertainty is based on several calibrations of a reference Schmidt-Boelter sensor.

An additional source of uncertainty, not present in open sensor calibration, arises in ellipsoidal radiometer calibration due to sharp gradients observed in radiometer output for small departures from the 0° orientation. The magnitude of this uncertainty, based on an alignment accuracy of ±0.1°, is about 1.1 %. The total relative expanded uncertainty is 3 % for a coverage factor *k* = 2 [7].

## 7. Conclusion

An ellipsoidal radiometer was characterized to determine the angular response, responsivity and purge gas effects, under narrow view-angle conditions, in the 25 mm variable temperature blackbody facility. Angular response measurements showed large gradients in the response in the angle range ±40° when the radiometer was rotated about a vertical axis passing through the aperture center. Also, the response distribution was observed to be anti-symmetrical with respect to 0° orientation. This behavior is possibly due to non-uniform reflection from top and bottom halves of the radiometer cavity surface. Also, it is speculated that a local defective spot on the gold-plated cavity surface might be a contributory factor, which can be easily corrected. The responsivity of the instrument, measured at 0° orientation, was found to be a function of its location from the blackbody. A decrease in responsivity by about 10 %, followed by a gradual increase was observed. This variation is believed to be largely due to the narrow view-angle calibration method rather than the radiometer, which is primarily designed for calibration and application under hemispherical viewing conditions. The angular response and the responsivity results are expected to be helpful in guiding the development of a newer version of the instrument for application in fire test methods. The effect of increasing purge-gas flow rate was to lower the signal output nearly linearly. The experimental results suggest that for flow rates less than 0.001 m^3^⋅s^–1^, the decrease in the radiometer signal output will be less than 1 %.

## Figures and Tables

**Table 1:** Transfer calibration results of ellipsoidal radiometer

Temperature	Distance		Linear regression	
K	*d*	Responsivity	Intercept	*R*-square
1573 K–2273 K	0.132	1.422 mV⋅(kW⋅m^–2^)^–1^	–0.004 mV	1.000
1573 K–2873 K	0.221	1.343 mV⋅(kW⋅m^–2^)^–1^	–0.002 mV	1.000
2773 K^a^	0.268	1.295 mV⋅(kW⋅m^–2^)^–1^	NA	NA
2073 K–2973 K	0.323	1.355 mV⋅(kW⋅m^–2^)^–1^	–0.003 mV	1.000
2773 K^a^	0.684	1.487 mV⋅(kW⋅m^–2^)^–1^	NA	NA

a Corresponds to angular response measurement conditions.

**Table 2:** Uncertainty in transfer calibration (%)

Uncertainty source	Type	Uncertainty
a. Transfer standard ESR (Previous calibration)	B	0.6
b. Blackbody temperature	B	0.1
c. Blackbody emissivity	B	0.0
d. Blackbody aperture uniformity	B	0.0
e. ESR & sensor alignment in VTBB–linear	B	0.4
f. ESR & sensor alignment in VTBB–angular	B	0.1
g. Radiometer averaging effect	B	0.1
h. ESR reading	A	0.1
i. Sensor reading	A	0.1
j. Long term repeat tests on a reference sensor	B	0.7
h. Radiometer output	B	1.1
Relative expanded uncertainty (*U* = *ku*_c_)	*k* = 2	3.0

## Footnotes

1 Also known as Gunners’ radiometer, named after the inventor.

2 Swedish National Testing and Research Institute, Sweden.
